# Supplementary material for: Role and mechanism of NCAPD3 in promoting malignant behaviors in gastric cancer
Source: Front Pharmacol. 2024 Apr 22;15:1341039. doi: 10.3389/fphar.2024.1341039 (PMC11070777; doi:10.3389/fphar.2024.1341039)
Supplement: Supplementary file 11 [file DataSheet2.ZIP › GSEA/GO_CC/my_analysis.Gsea.1599461408869/GO_VESICLE_LUMEN.html]

Details for gene set GO\_VESICLE\_LUMEN[GSEA]

|  || Dataset | filtered\_dataset.sample\_info.cls#WT\_versus\_NCAPD3\_MUT |
| Phenotype | sample\_info.cls#WT\_versus\_NCAPD3\_MUT |
| Upregulated in class | NCAPD3\_MUT |
| GeneSet | GO\_VESICLE\_LUMEN |
| Enrichment Score (ES) | -0.2938512 |
| Normalized Enrichment Score (NES) | -1.5720333 |
| Nominal p-value | 0.047263682 |
| FDR q-value | 0.12751631 |
| FWER p-Value | 0.805 |
Table: GSEA Results Summary

  

Fig 1: Enrichment plot: GO\_VESICLE\_LUMEN      
 Profile of the Running ES Score & Positions of GeneSet Members on the Rank Ordered List

  

| SYMBOL | TITLE | RANK IN GENE LIST | RANK METRIC SCORE | RUNNING ES | CORE ENRICHMENT || 1 | 2243 | FGA | 18 | 1.039 | 0.0496 | No |
| 2 | 5701 | PSMC2 | 244 | 0.636 | -0.0750 | No |
| 3 | 25852 | ARMC8 | 307 | 0.588 | -0.0844 | No |
| 4 | 2244 | FGB | 321 | 0.582 | -0.0588 | No |
| 5 | 5036 | PA2G4 | 365 | 0.557 | -0.0563 | No |
| 6 | 5718 | PSMD12 | 372 | 0.552 | -0.0274 | No |
| 7 | 387263 | C6orf120 | 400 | 0.535 | -0.0147 | No |
| 8 | 2919 | CXCL1 | 405 | 0.534 | 0.0146 | No |
| 9 | 3479 | IGF1 | 511 | 0.469 | -0.0331 | No |
| 10 | 178 | AGL | 711 | 0.368 | -0.1550 | No |
| 11 | 203068 | TUBB | 736 | 0.358 | -0.1508 | No |
| 12 | 23197 | FAF2 | 760 | 0.342 | -0.1468 | No |
| 13 | 3146 | HMGB1 | 831 | 0.295 | -0.1797 | No |
| 14 | 3312 | HSPA8 | 848 | 0.244 | -0.1765 | No |
| 15 | 6286 | S100P | 1011 | -0.412 | -0.2690 | Yes |
| 16 | 978 | CDA | 1023 | -0.421 | -0.2516 | Yes |
| 17 | 54676 | GTPBP2 | 1050 | -0.447 | -0.2435 | Yes |
| 18 | 1191 | CLU | 1056 | -0.450 | -0.2200 | Yes |
| 19 | 6590 | SLPI | 1062 | -0.453 | -0.1963 | Yes |
| 20 | 5834 | PYGB | 1096 | -0.475 | -0.1915 | Yes |
| 21 | 6709 | SPTAN1 | 1114 | -0.487 | -0.1745 | Yes |
| 22 | 6947 | TCN1 | 1135 | -0.503 | -0.1586 | Yes |
| 23 | 5154 | PDGFA | 1191 | -0.566 | -0.1643 | Yes |
| 24 | 7057 | THBS1 | 1242 | -0.620 | -0.1632 | Yes |
| 25 | 1893 | ECM1 | 1250 | -0.637 | -0.1298 | Yes |
| 26 | 1956 | EGFR | 1266 | -0.657 | -0.1011 | Yes |
| 27 | 7414 | VCL | 1308 | -0.712 | -0.0878 | Yes |
| 28 | 1116 | CHI3L1 | 1339 | -0.777 | -0.0627 | Yes |
| 29 | 7422 | VEGFA | 1346 | -0.788 | -0.0196 | Yes |
| 30 | 2444 | FRK | 1396 | -1.082 | 0.0101 | Yes |
Table: GSEA details [plain text format]

  

Fig 2: GO\_VESICLE\_LUMEN      
 Blue-Pink O' Gram in the Space of the Analyzed GeneSet

  

Fig 3: GO\_VESICLE\_LUMEN: Random ES distribution      
 Gene set null distribution of ES for **GO\_VESICLE\_LUMEN**
